# Possible antiviral effect of ciprofloxacin treatment on polyomavirus BK replication and analysis of non-coding control region sequences

**DOI:** 10.1186/1743-422X-10-274

**Published:** 2013-09-03

**Authors:** Ilaria Umbro, Elena Anzivino, Francesca Tinti, Assunta Zavatto, Anna Bellizzi, Donatella Maria Rodio, Carlo Mancini, Valeria Pietropaolo, Anna Paola Mitterhofer

**Affiliations:** 1 Department of Clinical Medicine, Nephrology and Dialysis Unit, “Sapienza” University of Rome, Viale dell’Università 37, Rome 00185, Italy; 2 Department of Public Health and Infectious Diseases, “Sapienza” University of Rome, Rome Italy; 3 Sbarro Institute for Cancer Research and Molecular Medicine, Center for Biotechnology, College of Science and Technology, Temple University, Philadelphia, Pennsylvania, USA

**Keywords:** Polyomavirus BK infection, Fluoroquinolones, Ciprofloxacin, Renal transplant, Acute renal dysfunction, Non-coding control region, Viral protein 1

## Abstract

Acute renal dysfunction (ARD) is a common complication in renal transplant recipients. Multiple factors contribute to ARD development, including acute rejection and microbial infections. Many viral infections after kidney transplantation result from reactivation of “latent” viruses in the host or from the graft, such as the human Polyomavirus BK (BKV). We report the case of a 39 year-old recipient of a 2^nd^ kidney graft who experienced BKV reactivation after a second episode of acute humoral rejection. A 10-day treatment with the quinolone antibiotic ciprofloxacin was administered with an increase of immunosuppressive therapy despite the active BKV replication. Real Time PCR analysis performed after treatment with ciprofloxacin, unexpectedly showed clearance of BK viremia and regression of BK viruria. During the follow-up, BK viremia persisted undetectable while viruria decreased further and disappeared after 3 months.

BKV non-coding control region sequence analysis from all positive samples always showed the presence of archetypal sequences, with two single-nucleotide substitutions and one nucleotide deletion that, interestingly, were all representative of the subtype/subgroup I/b-1 we identified by the viral protein 1 sequencing analysis.

We report the potential effect of the quinolone antibiotic ciprofloxacin in the decrease of the BKV load in both blood and urine.

## Background

Acute renal dysfunction (ARD) is a common complication in renal transplant recipients. Many different causes may lead to ARD such as acute rejection and microbial infections [1]. Most viral infections after renal transplantation result from reactivation of “latent” viruses in the host or from the graft, such as the human Polyomavirus BK (BKV) [2]. Multiple factors contribute to viral activation after transplantation, including immunosuppression, graft rejection therapy, inflammation and tissue injury [2]. Current antirejection therapy, based mainly on tacrolimus (Tac) and mycophenolic acid (MMF), has markedly decreased the incidence of acute rejection at the expenses of an increase of post-transplant BKV infections.

BKV has been identified as an important emerging cause of ARD, causing BKV nephropathy (BKVN) characterized by interstitial nephritis and/or urinary tract stenosis [3,4]. BKV nephropathy is an important complication after renal transplantation, affecting 1–10% of recipients and causing graft loss in approximately 50% of cases [5]. Immunosuppressive therapy and treatment for acute rejection are considered primary risk factors for BKVN [6-8]. Other factors associated with BKVN include donor and recipient characteristics, human leukocyte antigens (HLA) mismatching, ureteral stents, leukopenia, viral (co-)infections.

Limited therapeutic options are available against BKV and the mainstay of treatment in patients with BK viremia is reduction of immunosuppression [4,9-14]. This approach must be undertaken with caution because drug minimization or withdrawal strategies in immunosuppression can lead to acute rejection [15].

The present need to discover an efficacious treatment to prevent and manage BKV infection remains a priority particularly in transplant recipients with recurrent episodes of rejection as in highly sensitized patients.

Despite the lack of targeted antiviral intervention, a few agents have shown some anti-BKV activity, including cidofovir, leflunomide, intravenous immune globulins [16-18]. A number of *in vitro* and *in vivo* studies have demonstrated that fluoroquinolones are capable of inhibiting the helicase activity of BKV large T antigen (TAg) protein, which seems to be crucial for separation of the double-stranded DNA genome during replication [19-23]. However it is difficult to distinguish the specific effects of fluoroquinolones on BKV replication from those of a concurrent reduction of immunosuppression, which is recommended in these cases [24,25].

In this report we describe the case of a highly sensitized kidney re-transplant patient who needed an overall increase of immunosuppression, due to acute rejection, in course of ARD associated to BKV reactivation.

## Case presentation

A 39 year-old man developed two episodes of ARD 6 years after the second kidney transplant. He had a first kidney graft from his mother in 1993, which failed due to primary non-function. He continued renal replacement therapy and the allograft was not removed.

In 2004 he received the second kidney graft from a deceased donor. The DR1 HLA class II antigen was in common with the first kidney. Donor Specific Antibodies (DSA) before his second transplant were not detectable and the cross-match was negative as well. He received induction therapy with basiliximab at standard dosage and maintenance triple therapy with Tac (trough levels: 4–8 ng/mL), MMF and steroids. Serum creatinine remained stable for 3 years, with a range of 1.5-2 mg/dL.

In December 2007 he experienced one first episode of ARD, defined by an increase of serum creatinine above 25% of the baseline (3.6 mg/dL) and fever. BKV DNA was tested with Real-Time PCR (Q-PCR) in blood and urine and both samples resulted negative. The cross-match was mildly positive at <30%. He was empirically treated with steroid pulses and an overall increase in immunosuppression with a modest improvement of renal function.

**Figure 1:**
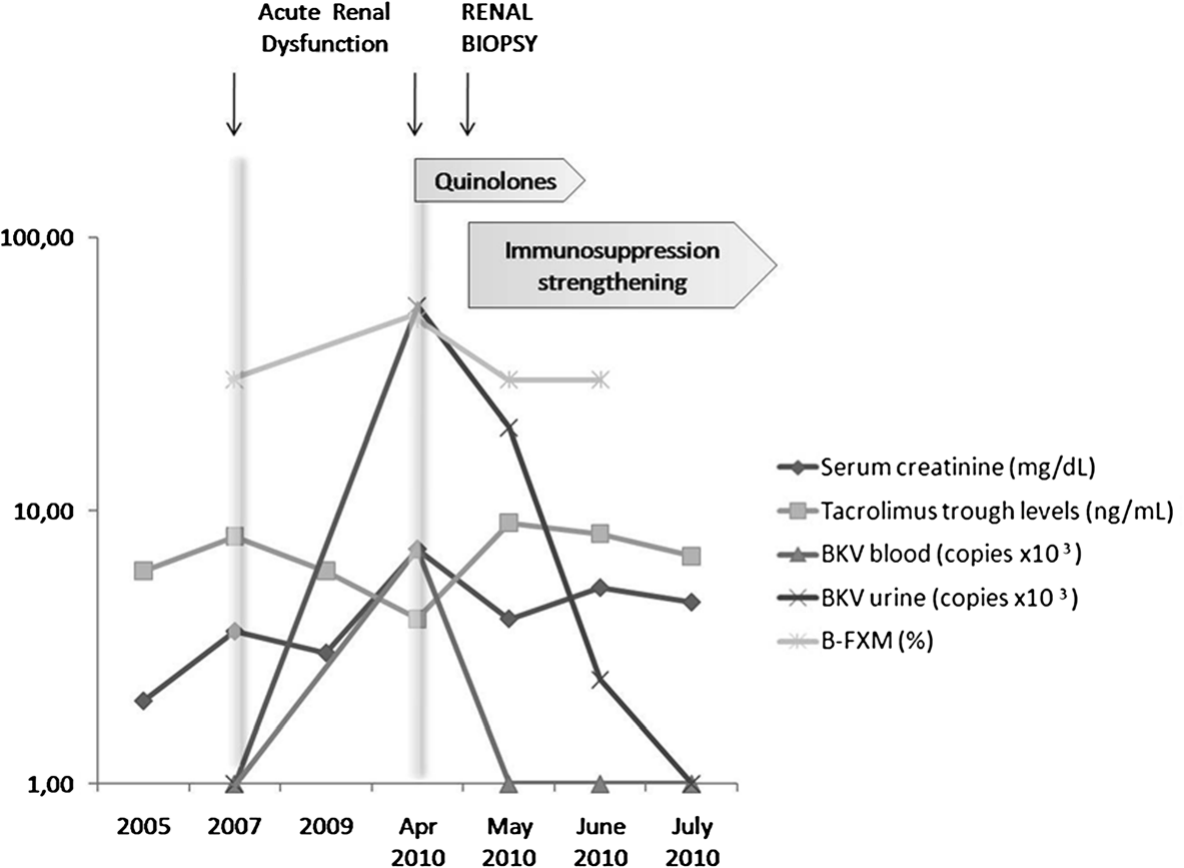
Time course of clinical events and laboratory examinations.

In April 2010 he developed a second episode of ARD, characterized by a further acute raise in serum creatinine to 7.2 mg/dL, reduction of diuresis, diffuse edematous state, proteinuria, metabolic acidosis and hypertension. At the same time BKV DNA, previously negative, converted both in blood and urine samples. BKV DNA loads were 7.2x10^3^ copies/mL and 5.6x10^4^ copies/mL, respectively (Figure 1); moreover B-cell cross-match (B-FXM) resulted positive (50%) and DSA against DR1 became detectable.

The transplanted kidney showed normal vascular patterns at Doppler ultrasound. A cystogram showed urethral stenosis and reflux at the uretero-vesical junction thus urinary tract infection prophylaxis was started using ciprofloxacin (250 mg twice daily for ten days), adjusted for glomerular filtration rate.

**Figure 2:**
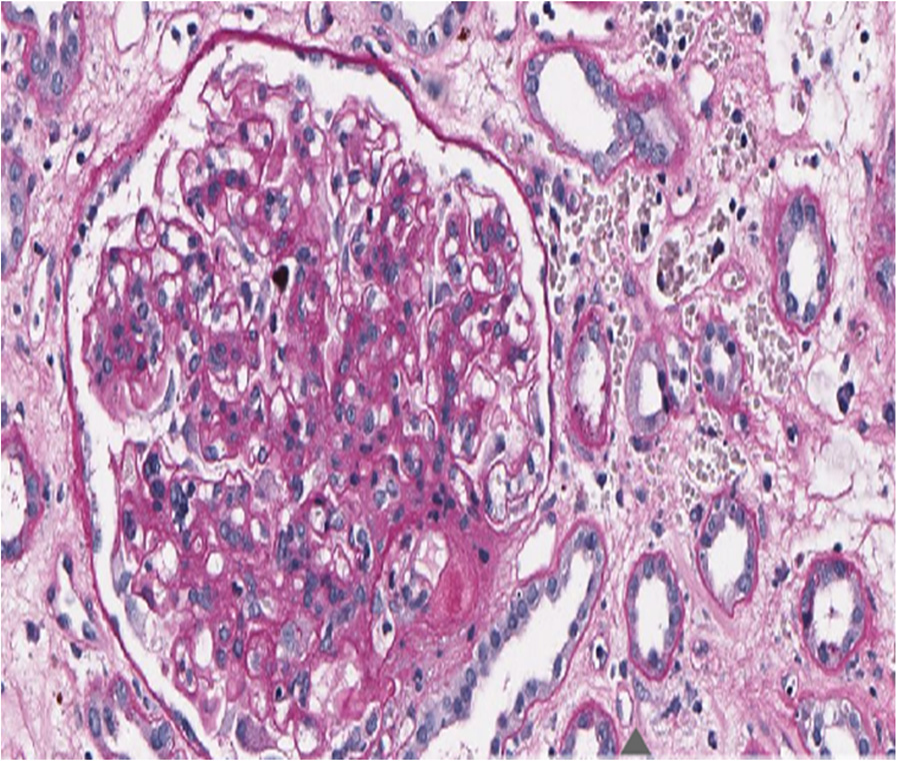
**Histopathological examination by Periodic Acid Schiff reaction, x200.** Glomeruli are characterized by widespread, intense mononuclear infiltration, moderate increase of the matrix and focal pericapsular fibrosis. Arteries show intense intimal fibrosis and fragmentation of the internal elastic lamina, arterioles are characterized by intense intimal hyalinosis. Edema and inflammatory infiltration of lymphocytes without plasmacells are present in the interstitium. Peritubular capillaries are markedly dilated with leucocytes margination. Epithelium of the proximal tubules occurs frequently flattened; into the lumen of the distal tubules, sometimes dilated, are contained hyaline casts. No nuclear inclusions are detected in the tubular epithelium. Search for C4d, evaluated by immunohistochemistry on tissue sections fixed, was positive (+ +) on the walls of peritubular capillaries.

The renal biopsy showed acute humoral rejection Banff type I [26] with no signs of BKVN (Figure 2). BKV detection by Q-PCR in renal tissue was negative. Therefore, in spite of active BKV replication, the immunosuppression was increased. The target Tac trough levels were set to 9 ng/mL and high dose steroid pulses and rituximab (375 mg/m^2^ weekly for 4 cycles) were given. Moreover he underwent 13 sessions of plasma-apheresis and 5 sessions of photo-apheresis.

BKV replication in blood and urine was strictly monitored. Real-Time PCR analysis performed in May, after treatment with ciprofloxacin, unexpectedly showed clearance of BK viremia and regression to 1x10^4^ copies/mL of viruria, despite the increase in immunosuppression. After a temporary mild improvement serum creatinine deteriorated further and was 5 mg/dL after 1 month. B-cell cross-match decreased but persisted positive (30%).

During the follow-up, BK viremia persisted undetectable while viruria further decreased (2.3x10^3^ copies/mL) until disappearing after 3 months. Unfortunately renal function did not improve, with hindsight probably due to the poor responsiveness of acute humoral rejection in the presence of irreversible chronic renal lesions. The patient started renal replacement therapy 5 months later.

Retrospectively we performed the molecular characterization of the BKV viral protein 1 (VP1) and non-coding control region (NCCR) on the urine and blood samples which were positive for viral DNA during the follow-up. Both regions, amplified by specific nested PCR and sequenced using a dedicated facility, were analysed to classify the BKV strains obtained into the corresponding subtype/subgroup, examining the single nucleotide polymorphisms within the VP1 region, and to investigate the presence of possible rearrangements within the NCCR. All BKV strains isolated in both urine and blood belonged to the archetype strains (WW), with VP1 genotype defined as subtype I/subgroup b-1.

## Discussion

We have reported the case of a highly sensitized re-transplant patient who developed a second ARD episode associated to BKV reactivation. The differential diagnosis between acute rejection and BKVN was crucial as these conditions require opposite therapeutic strategies [27]. Our patient underwent a renal biopsy which showed acute humoral rejection without signs of BKVN and Q-PCR analysis did not detect the virus in kidney cells. Consequently the moderate BKV load found in urinary and circulatory compartments could be due to a viral replication restricted to urothelial cells, as supported by the presence of urethral stenosis with active reflux of the uretero-vesical junction. Moreover BKVN can be focal in distribution, therefore kidney biopsy could fail to detect viral tissue replication [28].

Despite the active BKV replication the diagnosis of acute rejection led to an increase of the anti-rejection therapy.

Antiviral drugs to treat BKV were not commenced due to their nephrotoxicity [29], though BKV replication rate was strictly monitored in blood and urine. An unexpected reduction in BKV replication was noted. Plasma-apheresis played no role as it started after BK viruria regression to 1x10^4^ copies/mL. It was then hypothesized that fluoroquinolone ciprofloxacin, used as from the centre’s protocol for urinary tract infection prophylaxis after a cystogram, could have played a role in reducing viral replication.

It is known that quinolones display anti-BKV properties through inhibition of polyomavirus associated TAg helicase activity [19,20,24]. Fluoroquinolones not only reduce BKV DNA replication and the associated protein expression (for example TAg, viral capsid proteins and agnoprotein) but also lower the cell release of viral progeny by more than 90% [19]. Recently this class of antibiotics has been shown to have effects on BK viremia and/or viruria after transplantation; however all studies described the impact of prophylactic administration of these antibiotics on BKV replication or their association with simultaneous immunosuppression reduction [15,16,22,24,25,30]. In such cases it is difficult to know whether the reduction of BKV replication is due to a direct effect of fluoroquinolones or to the reduction of immunosuppression.

Real-Time PCR analysis performed after treatment with ciprofloxacin, unexpectedly showed a regression in BK viruria and the clearance of viremia. The decreasing trend in BKV load in urine was observed until July 2010 when BKV DNA disappeared. During the entire follow-up for BK viruria and viremia serum creatinine had a temporary slight improvement and Tac trough levels were maintained in therapeutic range (Figure 1). Unfortunately renal function did not improve and patient started renal replacement therapy 5 months later. We exclude a ciprofloxacin nephrotoxic role in renal function worsening because the renal biopsy showed the presence of irreversible chronic renal lesions not related to the drug toxicity. Moreover the drug dosage was adjusted for glomerular filtration rate.

**Figure 3:**
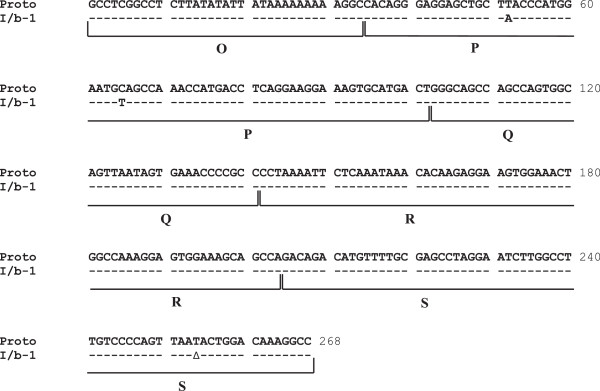
**Alignment of BKV prototypic NCCR sequence with archetypal WW NCCR sequences detected in our patient.** The prototypic (proto) NCCR is shown at the top of the figure and the BKV archetype WW strain NCCR consensus sequence (GenBank accession no. AB211371), belonged to subtype I subgroup b-1, we found in urine and blood specimens is shown below in relation to the prototypic NCCR, with similar nucleotides indicated by hyphens and the deletion identified by a triangle. Blocks (O, P, Q, R and S) commonly used to denote archetypal NCCRs [35] are indicated below the prototypic NCCR sequence.

To our knowledge, none of the studies reported in literature concerning the effects of fluoroquinolones on BKV replication has evaluated the presence of possible rearrangements within the BKV NCCR, a region of approximately 400 bp containing the origin of DNA replication (Ori) and the promoter/enhancer elements involved in transcriptional regulation of both the early and the late viral genes. BKV variants with rearranged NCCR have been identified in various studies including kidney transplant recipients [31]. Alignment of all NCCR sequences, detected from each BKV-positive clinical sample of our patient, with the prototypic NCCR sequence proposed by Yogo and colleagues [32], revealed that all specimens were defined by the presence of two nucleotide substitutions and a single nucleotide deletion occurring at nucleotide positions 52, 65 and 254, respectively (Figure 3). All these point mutations were typical of the consensus NCCR sequence identified for the BKV subtype I subgroup b-1 [32], which is widespread throughout the world [33]. The subgroup I/b-1 was also identified by molecular analysis of the BKV VP1 coding sequence between nucleotides 1744 and 1812 (amino acids 61 to 83) [34], which allows to define the BKV genotypes and their different distribution in the human populations.

Although NCCR is the most hyper-variable region of Polyomavirus genome, it still remains unclear what molecular mechanisms operates to generate rearranged NCCRs [36]. It has been suggested that recombination occurs between two newly synthesized daughter segments of a replicating DNA molecule at non-homologous points [37]. BKV genome is replicated bi-directionally starting from the Ori and the separation of daughter molecules is a slow step involving replicative intermediates with at least three chains, two daughter strands and the unreplicated parental DNA [38]. At the initial stage of replication, the three Ori sequences are in very close proximity, and this may facilitate major rearrangements through self-recombination. The lack of rearrangements within the non-coding control region of BK virus in both urinary and circulatory compartments of our patient may have depended on the mechanism of action of fluoroquinolones. As already mentioned above, these drugs represent a potent group of antibiotics able to inhibit BKV DNA replication by interfering with the helicase function of its TAg protein, which binds to the origin of replication of BKV DNA and coordinates its bidirectional replication in the presence of host DNA polymerase and topoisomerase II. It has been speculated that quinolones may prevent the TAg helicase activity by disrupting TAg-DNA interaction, due to a direct binding of these drugs to the DNA that TAg uses as substrate, or, alternatively, by converting the TAg-DNA complex into a frozen DNA-quinolone-TAg intermediate [20]. Nevertheless, whatever the exact mechanism of inhibition of TAg functions, the viral DNA cannot be replicated and consequently it cannot undergo rearrangements. Moreover, whether deletions and duplications of certain NCCR regions were associated with replicative advantage in an immunocompromised host, these alterations could be detected more frequently since they would be positive selected, but this did not happen in our case.

## Conclusions

This is the first report showing a progressive reduction in BKV replication in a patient who underwent ciprofloxacin treatment concurrently with an increase of immunosuppressive therapy.

The quinolone antibiotic ciprofloxacin administration could have played an active role in reducing BKV replication and eradicated the BKV load in both urinary and circulatory compartments.

## Consent

Written informed consent was obtained from the patient for publication of this Case Report and any accompanying images. A copy of the written consent is available for review by the Editor-in-Chief of this journal.

## Abbreviations

ARD: Acute renal dysfunction; BKV: Polyomavirus BK; Tac: Tacrolimus; MMF: Mycophenolic acid; BKVN: BKV nephropathy; HLA: Human leukocyte antigens; TAg: T antigen; DSA: Donor specific antibodies; Q-PCR: Real time PCR; B-FXM: B-cell cross-match; VP1: Viral protein 1; NCCR: Non-coding control region.

## Competing interests

The authors declare that they have no competing interests.
